# Molecular docking assisted exploration on solubilization of poorly soluble drug remdesivir in sulfobutyl ether-tycyclodextrin

**DOI:** 10.1186/s41120-022-00054-5

**Published:** 2022-04-25

**Authors:** Yumeng Zhang, Zhouming Zhao, Kai Wang, Kangjie Lyu, Cai Yao, Lin Li, Xia Shen, Tengfei Liu, Xiaodi Guo, Haiyan Li, Wenshou Wang, Tsai-Ta Lai

**Affiliations:** 1 Zhejiang Huahai Pharmaceutical Co., Ltd., Linhai, Zhejiang 317024 China; 2 METiS Pharmaceuticals, Inc., Hangzhou, Zhejiang 310052 China

## Abstract

**Objective:**

To study structure-specific solubilization effect of Sulfobutyl ether-β-cyclodextrin (SBE-β-CD) on Remdesivir (RDV) and to understand the experimental clathration with the aid of quantum mechanics (QM), molecular docking and molecular dynamics (MD) calculations.

**Methods:**

The experiment was carried out by phase solubility method at various pH and temperatures, while the concentration of Remdesivir in the solution was determined by HPLC. The complexation mechanism and the pH dependence of drug loading were investigated following a novel procedure combining QM, MD and molecular docking, based on accurate pKa predictions.

**Results:**

The phase solubility and solubilization effect of RDV in SBE-β-CD were explored kinetically and thermodynamically for each assessed condition. An optimal drug / SBE-β-CD feeding molar ratio was determined stoichiometrically for RDV solubility in pH1.7 solution. The supersaturated solubility was examined over time after pH of the solution was adjusted from 1.7 to 3.5. A possible hypothesis was raised to elucidate the experimentally observed stabilization of supersaturation based on the proposed RDV Cation A /SBE-β-CD pocket conformations.

**Conclusion:**

The computational explorations conformed to the experimentally determined phase solubilization and well elucidated the mechanism of macroscopic clathration between RDV and SBE-β-CD from the perspective of microscopic molecular calculations.

**Graphical Abstract:**

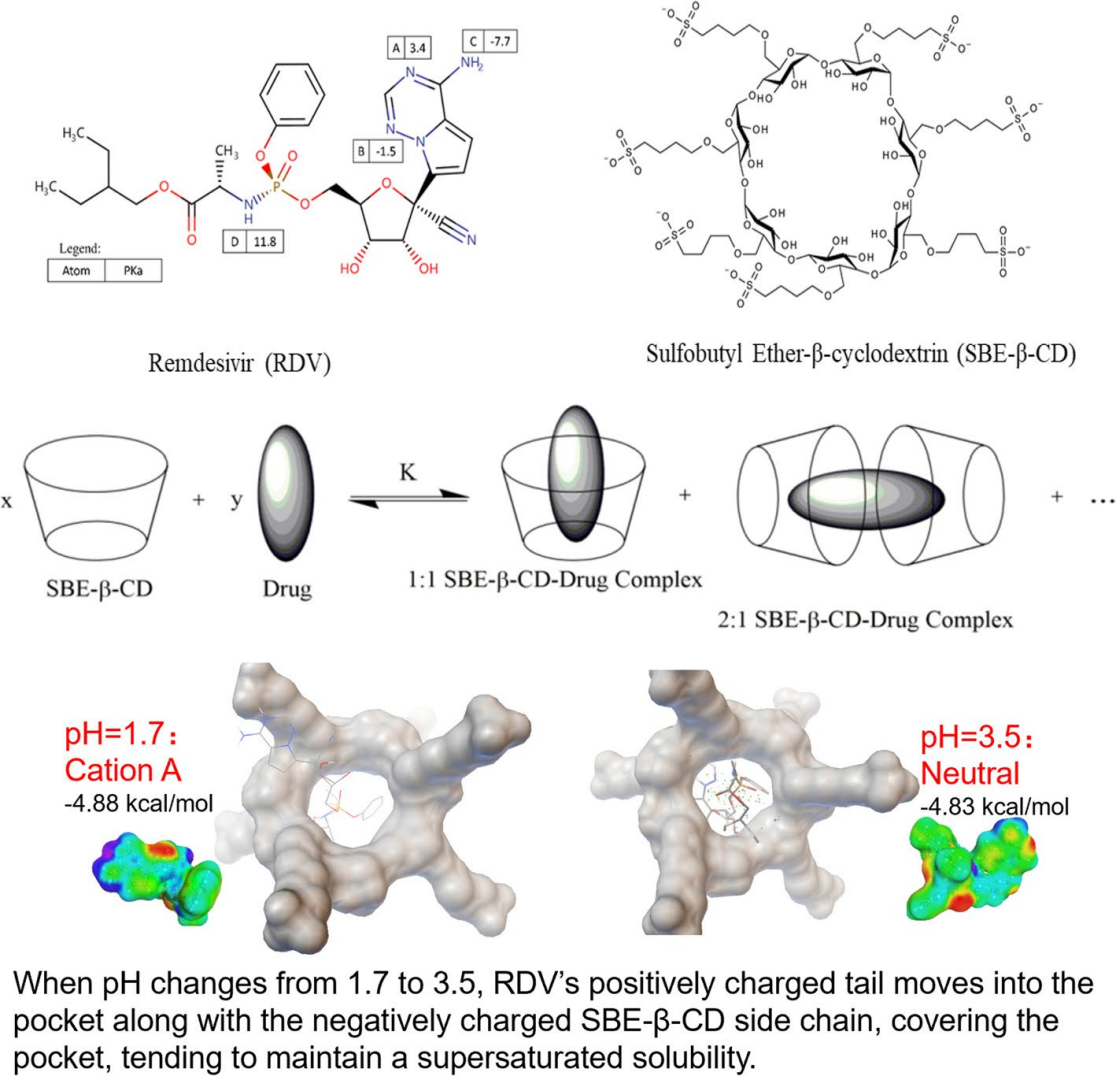

## Introduction

Remdesivir (RDV, GS-5734) is a monophosphamide prodrug of an adenosine analog with demonstrated antiviral activity against an array of RNA virus families including filoviridae, paramyxoviridae, pulmoviridae, and CoV (Brown et al. 2019), which indicates that RDV has more extensive medical use (Warren et al. 2016). There is patent (Larson and Saint 2019) information showing that RDV can be administered parenterally to some patients. However, RDV is relatively insoluble and chemically unstable in aqueous media, so it is necessary to develop a composition containing RDV, or a pharmaceutically acceptable salt thereof, that exhibits improved solubility, improved usability for parenteral administration, and sufficient room-temperature and elevated temperature stability to avoid the use of cold-chain for transport and/or storage. In addition to the active ingredients, the intravenous injection in the patent also contains cyclodextrin and pH regulator.

Cyclodextrin (CD) and its derivatives have the characteristics of hydrophilic outer edge and hydrophobic inner cavity (Carneiro et al. 2019; Jansook et al. 2018; Muankaew and Loftsson 2018; Kulkarni and Belgamwar 2017; Sherje et al. 2017; Pillai et al. 2017; Sharma and Baldi 2016; Kurkov and Loftsson 2013; Luke et al. 2010; Astray et al. 2009). Therefore, it can provide a hydrophobic binding site, as the main body to encapsulate all kinds of hydrophobic guest molecules, and form inclusion complexes by non-covalent bond. Sulfobutyl ether-β-cyclodextrin (SBE-β-CD) is a new kind of anion high water-soluble cyclodextrin derivative, C_42_H_70-n_O_35_(C_4_H_8_SO_3_Na)_n_. Compared with cyclodextrin, SBE-β-CD is more water-soluble and has higher in vivo safety (Sebestyén et al. 2013). SBE-β-CD is used as solubilizer of injection, without adding organic solvent, so as to improve the solubility and dissolution rate of the drug during solution preparation. At the same time, there is no residual problem of organic solvent after freeze-drying, which is conducive to safe drug use (Luke et al. 2010).

The last decades have witnessed the exceedingly rapid development of processing power and simulation algorithms. Simulation methods have long been used to accelerate the drug discovery process. In recent years, researchers have successfully applied simulation methods to drug formulation and drug delivery processes, including molecular docking, molecular dynamics (MD), and quantum mechanics (QM) simulations (Kulkarni and Belgamwar 2017; Weinzinger et al. 2007). In our study, it is important to evaluate the complexation mechanism of RDV and SBE-β-CD at different pHs. The key is to calculate the pKa values at different protonation states. To accurately predict pKa computationally, researchers (Pereira and Ramabhadran 2020; Casasnovas et al. 2014; Dutra et al. 2021) often use thermodynamic cycles that combine solvation free energies calculated with continuum solvent model and high level gas phase deprotonation free energies. However, most of these approaches have only been employed on small molecule datasets. COnductor like Screening MOdel for Real Solvents (COSMO-RS) is a promising theory in calculating various of properties, including pKa (Eckert and Klamt 2002; Klamt et al. 2003). Three out of four COSMO-RS-related methods have lower root mean square error (RMSE) than other QM-based methods in the recent overview of SAMPL6 pKa challenge (Işık et al. 2021). A result of RMSE lower than 1 pKa unit could be achieved.

In this study, the phase solubility and solubilization of RDV in sulfobutyl-β-cyclodextrin was investigated kinetically, thermodynamically, and stoicmetrically, where an optimal clathration condition was developed specific to the structures. To understand how and why the complexation progresses at different conditions, a novel procedure combining QM, MD, and molecular docking has been explored and applied. In order to determine the specific protonation states at different conditions, QM-based pKa calculations were performed.

## Experimental methods

### Materials and equipment

Remdesivir (received from Zhejiang Huahai Pharmaceutical Co., Ltd.), sulfobutyl ether-β-cyclodextrin (purchased from Zibo Qianhui Biological Technology Co., Ltd.), hydrochloric acid (purchased from Xilong Scientific Co., Ltd.), sodium hydroxide (purchased from Lin'an Qingshan Chemical Reagents Factory), water (Milli-Q grade, in-house), HPLC (Model e2695/2489, purchased from waters), column (C18, 150 × 4.6 mm, 3.5 μm, purchased from waters), pH meter (Model S400, purchased from Mettler Toledo), thermostatically controlled oscillator (purchased from Shanghai Yiheng Instrument manufacturing Co., Ltd.), shaker (purchased from Changzhou GW Instrument manufacturing Co., Ltd.), and centrifugator (purchased from Hunan Xiangyi Laboratory Instrument Company)

### HPLC analysis of RDV content of the complex

#### Mobile phase

Mobile phase A: 20 mM phosphate buffer, adjust the pH value to 5.0 ± 0.05 with NaOH solution (150 mM).

**Table:** Mobile phase B: acetonitrile

Time (min)	Mobile phase A (percent V/V)	Mobile phase B (percent V/V)
0–15	65 → 60	35 → 40
15–18	60 → 25	40 → 75
18–23	25	75
23–25	25 → 65	75 → 35
25–30	65	35

Flow rate: 1.0 ml/min

Detection wavelength: 245 nm

Injection volume: 10 μL

Column temperature: 35 ^o^C

The method was validated suitable for analysis of RDV in a linearity concentration range of 0.087 mM ~ 0.26 mM, with accuracy recovery of 98.0% ~ 102.0% and repeatability RSD < 3.0%.

### Phase solubility

#### Effect of pH on complexation of RDV in SBE-β-CD at different temperatures

SBE-β-CD was added into aqueous solution in different amounts to produce SBE-β-CD solutions with concentrations of 0, 11.56, 23.12, 46.24, 92.48, and 184.95 mM respectively. Prepare in 6 replicate and set each replicate of solutions at a pH. The pH was adjusted to 1.5, 1.7, 2.0, 3.0, 3.5, and 4.0 respectively using dilute hydrochloric acid aqueous solution. Excess amount of RDV was added into the above solutions in capped tubes and was shaked on a shaker for about 15 h at 25 °C. Thereafter, the suspensions were centrifuged at 10000 rpm, the supernatants were taken and diluted properly for HPLC analysis of RDV concentrations. The HPLC sample temperature was set at the study temperature. The same experiment was repeated for all sampe concentrations at 37 °C and 45 °C, respectively.

#### Effect of incubation time on complexation of RDV in SBE-β-CD

Following the same procedure in “Effect of pH on complexation of RDV in SBE-β-CD at different temperatures” section, SBE-β-CD aqueous solutions (0.0–185 mM) were prepared and pH was adjusted to 1.7. Excess amount of RDV was added into the solutions in capped tube and was shaked on a shaker at 25 °C. The solutions were sampled at scheduled time, centrifuged at 10,000 rpm, and the supernatants were taken and diluted properly for HPLC analysis of RDV concentration. The sampling times were 0.5 h, 1 h, 2 h, 3 h, and 15.5 h.

#### Effect of feeding molar ratio on complexation of RDV in SBE-β-CD

Seven different Drug/SBE-β-CD molar ratios (1:16, 1:10, 1:8, 1:7, 1:5, 1:4, 1:2) were selected for solubilization feeding in solutions with SBE-β-CD concentration of 92.57 mM. The temperature and pH of the solutions were controlled at 25 °C and 1.7. The obtained solutions were centrifuged at 10,000 rpm and the supernatants were diluted properly for HPLC analysis of RDV concentrations. The effect of different feeding molar ratios on the solubility of RDV was investigated to determine the optimal molar ratio for solubilization.

#### Stablization of complexation of RDV in SBE-β-CD

Excess amount of RDV exceeding its solubility was added to HCl buffer solution (pH1.7) containing SBE-β-CD, (92.5 mM) in capped tube, shaked on a shaker for about 15 h at 25 °C. Thereafter, adjusted pH of the solution from 1.7 to 3.5 and mixed thoroughly. The adjusted solution was sampled at scheduled time points, centrifuged at 10,000 rpm, the supernatant was taken and diluted properly for HPLC analysis of RDV concentration.

## Computational methods

### Structure preparation

The RDV structure was obtained from PubChem. The SBE-β-CD structure was manually modified from the β-CD structure obtained from Protein Data Bank. Six sulfobutyl ether groups were grafted onto the primary hydroxyl groups (6-OH on the glucose subunit). This was in accordance with the structure used in the experimental studies.

### Conformation analysis

To increase the docking accuracy, conformation analysis for both SBE-β-CD and RDV was performed. Different procedures were taken for SBE-β-CD and RDV due to their size difference and hence the computational cost.

MD simulations and energy minimization were performed using xTB (Grimme et al. 2017) and 2000 initial configurations were generated. Molclus (Lu 2020) program was then used for clustering and energy calculations calling ORCA (Neese 2018; Neese 2012). Fifteen representative configurations with lowest energies were chosen and optimized at BP-D3(BJ)/def-SVP (Schäfer et al. 1994; Becke 1993; Becke 1988; Perdew 1986; Goerigk and Grimme 2011) level of theory. Ten lowest-free-energy configurations were subsequently chosen for further high-accuracy QM optimization at (BP-D3(BJ)/def-TZVP) (Eichkorn et al. 1997) level of theory. COSMO files as inputs for pKa calculations were calculated in TURBOMOLE (Ahlrichs et al. 1989). The SMD implicit solvation model (Marenich et al. 2009) of water was used for all ionic species. Only one BP-D3(BJ)/def-SVP optimized structure was calculated for the larger SBE-β-CD.

### pKa calculations

We found one reported pKa value for RDV, 3.3 (Gilead Sciences 2020), in the VELKURY description document by Gilead. We used this value as the reference in our study to compare with the QM-calculated pKa values. It is important to point out that the authors in that document didn’t provide any further details other than a single value. This was why we put in the efforts to calculate the pKa values using QM-based methods on our own other than simply using that value as the reference value.

Before any pKa calculations, we needed to determine the most stable neutral state. We calculated the energies of the neutral RDV and all possible RDV zwitterions, the state with the lowest energy was chosen as the starting point for all following calculations

### Solvation model based on density (SMD)

The pKa values were calculated using the direct method (Thapa and Schlegel 2017; Ho 2015) (which does not require the gas-phase optimization) using Eq. (1):

1 $$p{K}_{\mathrm{a}}=\frac{\Delta {G}_{\mathrm{a}\mathrm{q}}^{\ast }}{2.303 RT}$$

where $\Delta {G}_{\mathrm{aq}}^{\ast }$ is the Gibbs free energy difference of the deprotonation reaction in the solution, *R* and *T* are the ideal gas constant and temperature, respectively.

$\Delta {G}_{\mathrm{aq}}^{\ast }$ can be calculated by Eq. (Mirzaei et al. 2019) (2):

2 $$\Delta {G}_{aq}^{\ast }={G}_{\mathrm{aq}}^{\ast}\left({\mathrm{A}}^{-}\right)-{G}_{\mathrm{aq}}^{\ast}\left(\mathrm{HA}\right)+{G}_{\mathrm{aq}}^{\ast}\left({\mathrm{H}}^{+}\right)$$

where ${G}_{\mathrm{aq}}^{\ast}\left({\mathrm{A}}^{-}\right)$and ${G}_{\mathrm{aq}}^{\ast}\left(\mathrm{HA}\right)$ are the calculated free energies of deprotonated and protonated acids, ${G}_{\mathrm{aq}}^{\ast}\left({\mathrm{H}}^{+}\right)$ is the aqueous phase free energy of proton, which was set to − 270.29 kcal/mol (Mirzaei et al. 2019).

All geometry optimizations were performed at B3YLP-D3(BJ)/6-31 + G* (Francl et al. 1982a; Francl et al. 1982b; Hehre et al. 1972; Clark et al. 1983; Frisch et al. 1984) level of theory using the SMD implicit solvation model. More accurate gas single point energy calculations were performed at RI-PWPB95-D3(BJ)/def2-QZVPP (Goerigk and Grimme 2011; Eichkorn et al. 1997; Weigend 2006; Hellweg et al. 2007; Grimme et al. 2010; Kruse and Grimme 2012) level of theory in ORCA. A less accurate single point energy at M06-2X/6-31G** (Zhao and Truhlar 2008) level was also performed to determine what level of accuracy would be enough.

#### SMD model with three explicit water molecules

For more accurate pKa prediction for cation A, three water molecules were added to RDV cation A and neutral RDV, geometry optimizations were performed at B3YLP-D3(BJ)/6-31G* level of theory using the SMD implicit solvation model. Frequencies were examined to confirm stationary points and scaled by 0.9813.

#### COSMO-RS

The pKa values were estimated using COSMO-RS with a linear-free energy relationship (Klamt et al. 2003).

3 $$p{K}_{a\kern0.5em }^i={c}_0+{c}_1\left({G}_i^{\mathrm{neutral}}-{G}_i^{\mathrm{ion}}\right)$$

where *c*_0_ and *c*_1_ are fitting parameters, ${G}_i^{\mathrm{neutral}}$ and ${G}_i^{\mathrm{ion}}$ are the free energies calculated following COSMO-RS protocols for molecule in water at neutral and protonated states.

### Molecular docking calculations

AutoDock (Morris et al. 1998) 4.3 was used to study the complexation of RDV and SBE-β-CD. The deprotonated state of SBE-β-CD was used as the host molecule. The protonated states of interest for RDV were used as the guest molecules and were docked to SBE-β-CD; the host in this system. We used the deprotonation state for SBE-β-CD as we estimated the pKa value for SBE-β-CD to be lower than 0.

The MD- and QM-optimized conformations were used for RDV and SBE-β-CD. For RDV, the conformation with the lowest energy was used. SBE-β-CD was prepared according to the standard AutoDockTools protocols (Kitchen et al. 2004), hydrogens were added and Gasteiger partial charges were assigned. The AutoGrid default grid spacing was used, with the grid box size set to be the size that effectively covers the whole SBE-β-CD.

RDV was docked for five times to SBE-β-CD, which was kept rigid in the process. It is worth mentioning that, since we were using the QM-optimized structure originally based on the crystal structure (Schmidt et al. 1998) the docking process would represent in some ways the real scenario of the complexation process of RDV and SBE-β-CD. One-hundred poses were generated for each docking task, which were subsequently clustered based on their free energies. The best representative pose was then visualized and analyzed.

## Results and discussion

### Effect of pH on phase solubility of RDV in SBE-β-CD

The phase solubility of RDV in SBE-β-CD (S) were studied in solutions at pH of 1.5, 1.7, 2.0, 3.0, 3.5, and 4.0, respectively, within a wide SBE-β-CD composition ranging from 0 ~ 185 mM. The phase solubility diagrams were determined at 25 °C, 37 °C, and 45 °C as the data listed in Table 1, where S/S_0_ was also calculated to directly reflect the solubilizing effect and *S*_0_ was the solubility of RDV in the absence of SBE-β-CD. At those temperatures, for the same SBE-β-CD concentration, the solubilization of RDV *S*/*S*_0_ increases with decrease of pH, indicating enhanced complexation of SBE-β-CD with the alkaline API in acid environment. While in solutions at same pH, the solubility of RDV (*S*/*S*_0_) increases with increase of SBE-β-CD concentration, indicating enhanced solubilizing effect of SBE-β-CD on the drug. Specifically, the solubility of RDV increases linearly as a function of SBE-β-CD concentrations (< 46 mM) showing an *A*_L_ type diagram according to Higuchi and Connors (Brewster and Loftsson 2007; T. Higuchi 1965), and then increases nonlinearly at higher SBE-β-CD concentrations. The apparent stability constant of the inclusion complexes formed (*K*) was calculated according to

4 $$\mathrm{K}=\mathrm{slope}/\left[{\mathrm{S}}_0\left(1-\mathrm{slope}\right)\right]$$

where slope represents the linear slope of corresponding phase solubility diagram. Table 2 contains determined *K* values at pH of 1.5, 1.7, 2.0, 3.0, 3.5, and 4.0 at 25 °C, 37 °C, and 45 °C, respectively. Based on Gibbs and Van’t Hoff Eq. (5) and (6), by plotting lnK vs 1/T, ΔH, and ΔS can be obtained from the slope and intercept respectively.

5 $$\Delta \mathrm{G}=-\mathrm{RTln}\ \mathrm{K}$$

6 $$\mathrm{lnK}=-\Delta \mathrm{H}/\left(\mathrm{RT}\right)+\Delta S/R$$

**Table 1:** Phase solubilization of RDV in SBE-β-CD in different pH media

SBE-β-CD Conc (mM)	pH1.5		pH 1.7		pH2.0		pH3.0		pH 3.5		pH 4.0	
	S	S/S_0_	S	S/S_0_	S	S/S_0_	S	S/S_0_	S	S/S_0_	S	S/S_0_
0.00	1.735	1	1.369	1	**25 °C** 0.707	1	0.087	1	0.033	1	0.038	1
11.56	8.542	5	7.507	5	5.710	8	0.871	10	0.432	13	0.301	8
23.12	15.926	9	12.614	9	8.406	12	1.253	14	0.602	19	0.458	12
46.24	26.460	15	19.689	14	11.827	17	1.897	22	0.968	30	0.883	23
92.48	39.646	23	24.427	18	14.701	21	3.001	35	1.467	45	1.608	42
184.95	52.206	30	29.939	22	18.887	27	4.667	54	2.465	76	2.836	75
					**37 °C**							
0.00	2.355	1	1.521	1	0.745	1	0.084	1	0.046	1	0.035	1
11.56	9.250	4	8.573	6	5.869	8	1.130	14	0.505	11	0.369	11
23.12	16.117	7	13.357	9	8.996	12	1.498	18	0.721	16	0.616	18
46.24	26.754	11	23.641	16	11.983	16	2.077	25	1.272	28	1.102	32
92.48	44.248	19	30.012	20	14.280	19	3.071	37	2.330	51	2.130	62
184.95	59.824	25	42.636	28	17.663	24	5.983	72	4.744	104	4.377	127
					**45 °C**							
0.00	2.352	1	1.594	1	0.927	1	0.109	1	0.060	1	0.047	1
11.56	9.385	4	8.280	5	5.538	6	0.841	8	0.529	9	0.397	9
23.12	15.239	6	14.319	9	8.662	9	1.222	11	0.786	13	0.511	11
46.24	24.685	10	21.357	13	13.484	15	2.104	19	1.700	29	1.502	32
92.48	36.319	15	27.591	17	16.003	17	2.920	27	2.100	35	1.865	40
184.95	42.998	18	30.422	19	26.649	22	3.935	36	3.139	53	4.154	89

**Table 2:** Stability constants of RDV / SBE-β-CD inclusion complexes

pH	K(L·mol^-1^)@25 °C	K(L·mol^-1^)@37 °C	K(L·mol^-1^)@45 °C
1.5	151	189	107
1.7	64	85	68
2.0	45	39	50
3.0	76	53	70
3.5	76	168	83
4.0	224	292	129

The results in Table 3 show that Δ*G* of the inclusion process of RDV and SBE-β-CD in each studied pH medium is negative, indicating that the inclusion process is a spontaneous formation process. There are positive and negative values of Δ*H* under different pH conditions, meaning that the inclusion process under different pH conditions is either an endothermic reaction or an exothermic reaction. Except for the value at pH 4.0, the ΔS values at other lower pH or more acidic conditions are all positive, showing that the entropy values increase in this inclusion process and thus drive the inclusion complexation toward more stable formation.

**Table 3:** Inclusion thermodynamic parameters at different pH

pH	ΔG (KJ·mol^-1^ )			ΔH (J·mol^-1^ )	ΔS (J· mol^-1.^K^-1^)
	298K	310K	318K	298~318 K	298~318K
1.5	-12.43	-13.50	-12.35	-11039.7	5.58
1.7	-10.31	-11.46	-11.16	3809.7	47.91
2.0	- 9.44	- 9.45	-10.36	3037.4	41.43
3.0	- 10.74	- 10.23	-11.23	-5271.7	17.70
3.5	-10.73	-13.21	- 11.68	7810.4	63.76
4.0	-13.40	-14.63	- 12.85	-18071.0	-14.40

### Effect of temperature on phase solubility of RDV in SBE-β-CD

From the same results in Table 1, the effects of temperature on solubilization of RDV in SBE-B-CD are assessed from perspective of both the determined phase solubility (*S*) and *S*/*S*_0_. Considering potential variation may incur among the single replicate determined phase diagrams, *S*/*S*_0_ helps to reduce intra system error while the magnitude of each *S*/*S*_0_ trend is subject to the accuracy of *S*_0_, where examination on *S* is therefore necessary. Comparisons of the phase diagram data sets at each pH illustrate that when temperature rises from 25 °C to 37 °C, the phase solubility of RDV (*S*) increases slightly, and when temperature continues to rise from 37 °C to 45 °C, S/S_0_ tends to decrease at each studied pH though S is not consistent among the data sets. The results indicate that proper heating is conducive to improving the solubilizing effect of SBE-β-CD on RDV, while further elevated temperature is not favorable for the solubilization probably due to increasing competitive decomplexation.

### Effect of incubation time on phase solubility of RDV in SBE-β-CD

**Fig. 1:**
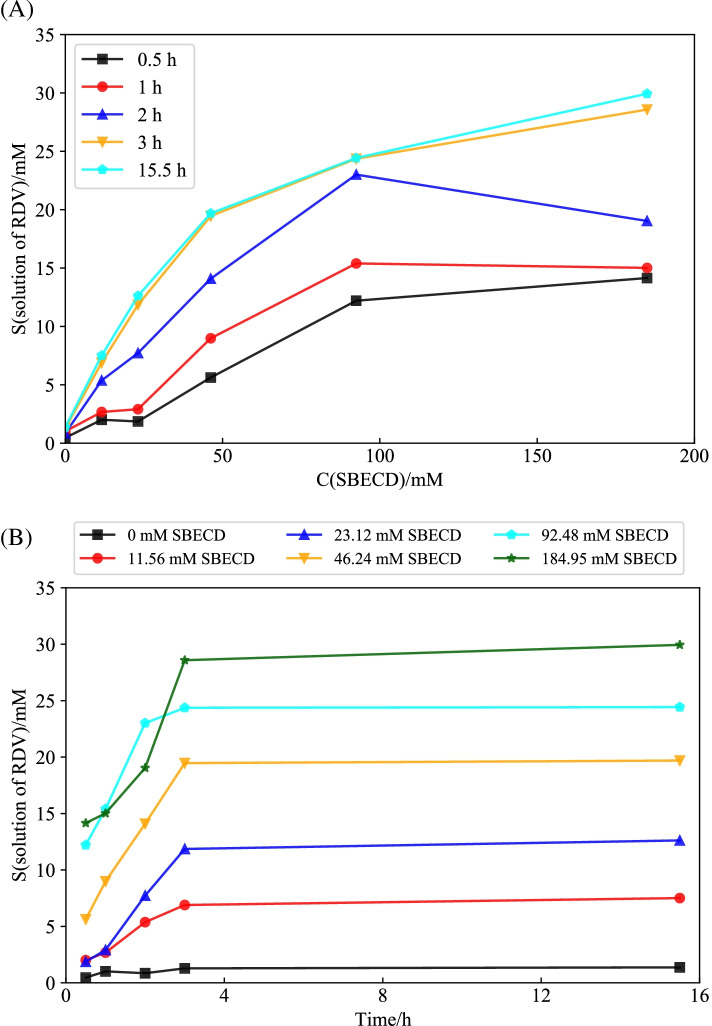
Effect of incubation time on phase solubility of RDV in SBE-β-CD in pH1.7 media at 25 °C: (**A**) S vs C; (**B**) S vs Time

Given the fact that solubilization of RDV in SBE-β-CD is in dynamic equilibrium, the effect of incubation time on phase solubility was investigated in pH 1.7 solution. From results in Fig. 1A, it is seen that each incubation time shows a unique phase solubility diagram though, in general, the solubilization effect increases with increase of incubation time. Specifically, for incubation time of 0.5, 1, and 2 h, the solubilization effect increases linearly as a function of SBE-β-CD concentration (up to 92.5 mM) following an *A*_L_ pattern while beyond that concentration, the solubilization effect tends to decrease at the higher SBE-β-CD concentration due to insufficient incubation time allowed for clathration to complete and agglormeration of SBE-β-CD occurred at higher concentration (Saokham et al. 2018). The formation of SBE-β-CD aggregates at the higher SBE-β-CD concentration may not only pull SBE-β-CD monomer away from chelation with RDV but also promote declathration of existing drug/SBE-β-CD complexes, leading to decreased phase solubility The competition between solubilization and declathration reaches a bottleneck maximum or inflection point at the incubation time of 2 h. When incubation time rises from 2 h to 3 h, the solubilization effect increases linearly as a function of SBE-β-CD concentration up to 46 mM and then increases nonlinearly until reaching equilibrium of RDV in SBE-β-CD. When the incubation time increases from 3 h to 15.5 h, there is basically no difference between the phase solubility as well as solubilization effect as shown in Fig. 1B.

### Effect of feeding molar ratio on phase solubility of RDV in SBE-β-CD

Following the previous discussion in “Effect of pH on phase solubility of RDV in SBE-β-CD” section, the saturated RDV phase solubility increases with increase of SBE-β-CD concentration or mass fraction, i.e, the saturated solubility increases with increase of SBE-β-CD mass fraction in the solutions. In practical product development applications, however, the molar ratio at the saturated solubility is typically not suggested for feeding use to avoid potential precipitation. In the current study, the effect of drug/SBE-β-CD feeding ratio on phase solubility was assessed in pH1.7 media at 25 ^o^C with SBE-β-CD concentration of 92 mM. It can be seen from Table 4 that with increase of drug /SBE-β-CD feeding molar ratio from about 1/16 to 1/7, the solubility of RDV first increases linearly. After that, the solubility of RDV increases nonlinearly, and then approaches saturation at around 1/4 molar ratio. When the molar ratio further increases from 1/4 to 1/2, the solubility of RDV reaches saturation with no significant increase. Based on the determined RDV concentrations, the actual molar ratios of drug/SBE-β-CD in solutions are also calculated as listed in Table 4. The molar ratios of drug/SBE-β-CD in solutions match the feeding molar ratios from 1/16 to 1/7. After that, the molar ratios in solutions are lower than the feeding ratios. Especially, when the feeding ratio goes up to 0.48, the actual molar ratio in solution is only 0.27, proving that the RDV solubility has reached saturation. Overall, in studied solutions, the unsaturated RDV phase solubility increases with increase of drug/SBE-β-CD feeding molar ratio until it reaches saturation.

**Table 4:** Effect of feeding molar ratio on solubility of RDV (pH=1.7, 25 °C)

Feeding molar ratio of RDV / SBE-β-CD (actual value)	S / (mM)	Determined molar ratio of RDV / SBE-β-CD in solution
1/16 (0.064)	5.680	0.061
1/10 (0.103)	9.205	0.099
1/8 (0.119)	10.741	0.116
1/7 (0.148)	13.402	0.145
1/5 (0.193)	14.264	0.154
1/4 (0.256)	22.916	0.248
1/2 (0.477)	24.645	0.266

### Stablization of complexation of RDV in SBE-β-CD

**Fig. 2:**
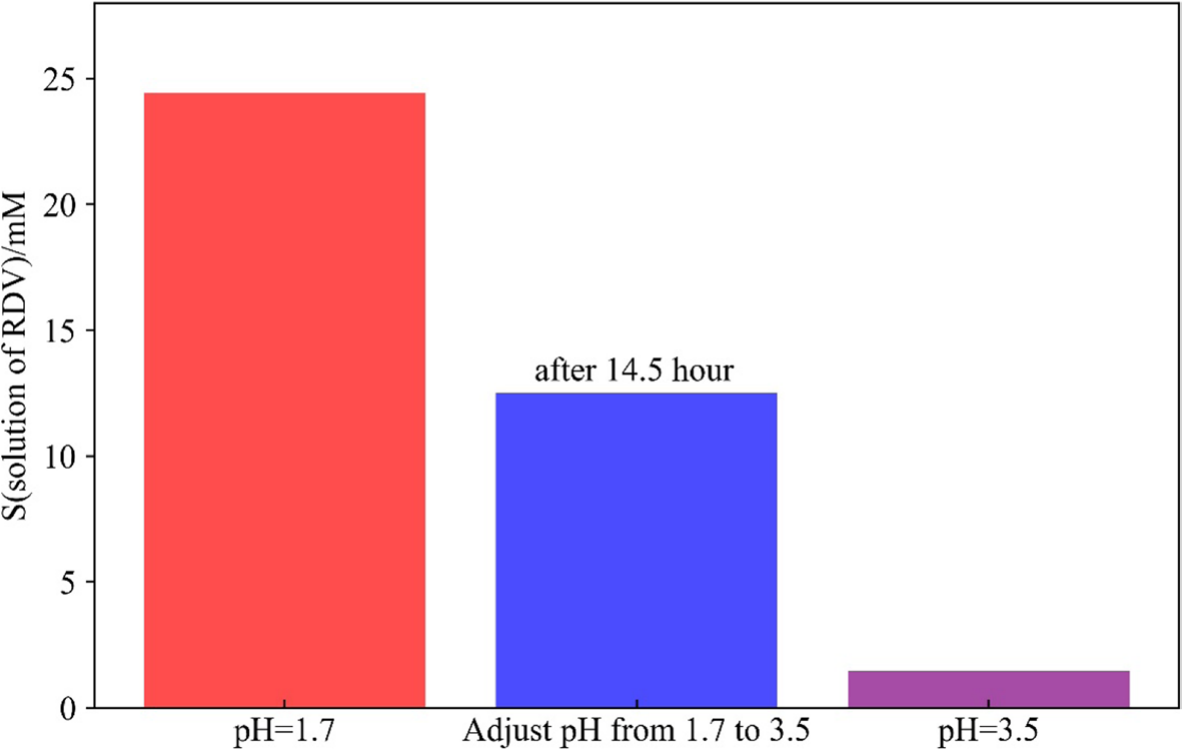
Stablization of supersaturated solubility of RDV in SBE-β-CD at 25 °C upon the adjustment from pH1.7 to pH3.5

As shown in Fig. 2, in 92.5 mM SBE-β-CD solution at 25 ^o^C, RDV has a solubility of 24.4 mmol/L at pH 1.7 while 1.5 mmol/L at pH 3.5, respectively. When pH of the complexation solution was adjusted from pH1.7 to pH3.5, however, instead of quickly dropping back to the level of 1.5 mmol/L, the solubility of RDV gradually decreased with time and maintained a stable concentration at 12.5 ~ 12.7 mmol/L when measured at 14.5 ~ 15.5 h after the pH adjustment, showing obvious stabilization of supersaturated solubility toward the pH adjustment, which be further discussed later with the aid of molecular docking conformations. The stabilization of supersaturation is an important feature in parenteral product manufacturing process.

### pKa calculations for RDV

Before any further calculations, the most stable neutral state was determined. As shown in Table 5, energies in a. u. and in kcal were calculated using TURBOMOLE. The neutral RDV has an energy lower than all the zwitterions and was used as the starting point for the following calculations.

**Table 5:** The lowest configuration energy of different neutral states

Structure	Energy(a.u.)	ΔE(a.u.)	ΔE(kcal)
RDV	-2322.53	0	0
Zwitterion_A	-2322.5	0.030717	19.27532
Zwitterion_B	-2322.49	0.035028	21.98072
Zwitterion_C	-2322.46	0.070013	43.93398

Based on our experimental conditions, we focused our calculations on determination of RDV acidic pKa. Starting from the neutral state, we listed all the possible protonation states, A-D, as labeled in RDV’s structrue in Graphical Abstract, where the energies for all four states were calculated and cation A was the most stable. We then calculated the pKa values of all four states using different QM-based methods. Table 6 shows the pKa values for cation A calculated by different methods. As shown, COSMO-RS gives the most accurate predictions. Adding waters increases the accuracy for the QM calculations, but error accumulated at different steps in the thermodynamic cycle which gives rise to inaccurate predictions. COSMO-RS-based method is more data-driven, it fitted the equation with experimental data at a more direct way, resulting to very accurate predictions for RDV. The generalizability of COSMO-RS in predicting pKa values for drugs and drug-like molecules has to be further examined, but this definitely points to a quicker, more direct, and more accurate way of predicting pKa values for drugs and drug-like molecules, especially for new molecules and molecules with high pH dependencies which would add difficulties to the experimentalists to measure pKa values.

**Table 6:** QM-based pKa values for Cation A

QM methods	pKa
(M06-2x/6-311G**)	-1.7
(M06-2x/6-311G**) with water	4.6
(RI-PWPB95-D3(BJ)/def2-QZVPP)	1.2
(RI-PWPB95-D3(BJ)/def2-QZVPP) with water	5.0
COSMO-RS	3.4
Experimental (Gilead)	3.3

### Docking of RDV to SBE-β-CD elucidates the complexation mechanism

**Fig. 3:**
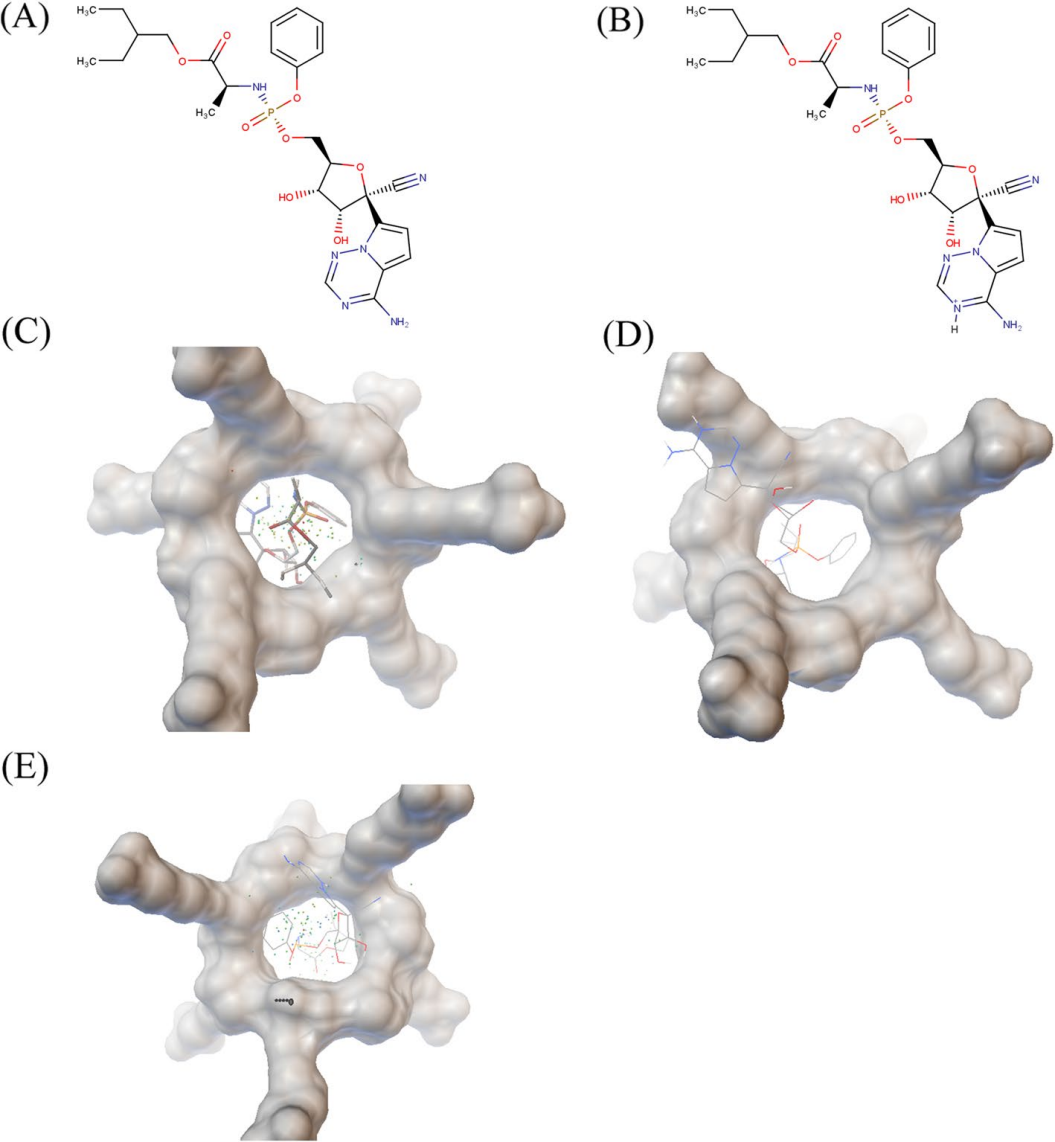
Docking poses of neutral RDV and RDV Cation1: (**A**) structure of neutral RDV; (**B**) structure of RDV Cation A; (**C**) docking pose of neutral RDV; (**D**) docking pose of RDV Cation A; (**E**) docking pose of RDV Cation B. The small dots in the docking poses represent the locations of the first 50 most stable poses during computations

The neutral RDV and the RDV cation A were docked to SBE-β-CD and the best representative poses were analyzed, as shown in Fig. 3. Figure 3A, B shows the structures for neutral RDV and RDV cation A. Figure 3 C–E are the most stable docking poses in SBE-β-CD (shown in van der Waals surfaces) for neutral RDV and RDV cations A and B, respectively. The small dots represent the locations of the first 50 most stable poses. For the case with neutral RDV, the guest was almost perfectly posed inside the SBE-β-CD pocket. However, for the case of RDV cation A, the pose was more extended. About half of the pose stayed in the pocket, with the other half, more specifically, the positively charged part was outside the pocket, close to the negatively charged sulfobutyl side chain. The RDV cation A pose (− 4.88 kcal/mol) has a lower binding free energy than the neutral RDV pose (− 4.83 kcal/mol), indicating that, although RDV was not fully inserted into the pocket, the electrostatic interaction makes up for the lost hydrophobic interaction. The two types of interactions both exist in the RDV cation A case. And the collective effect leads to a tighter complexation.

We also examined another RDV protonation state, the RDV cation B, a proton was added to the para-position of the amino group, instead of the ortho-position, as for RDV cation A. The most stable pose was shown in Fig. 3(E). The binding free energy of RDV cation B and SBE-β-CD (− 4.62 kcal/mol) was the biggest of the three, i.e, the least favorable. More interestingly, the positively charged part does not move towards the negatively charged sulfobutyl side chain, unlike the RDV cation A case. This finding further proves that the collective hydrophobic and electrostatic effect leads to a tighter binding for the RDV cation A to SBE-β-CD, tighter than the neutral RDV.

### Docking of RDV to SBE-β-CD elucidates the pH dependence of drug loading

As shown in Table 1, when pH decreases from 3.5 to 1.5, drug loading increases.

As mentioned above, the nitrogen in the ortho-position of the amino group has a pKa of 3.4. Upon simple calculations, at pH = 1.5, the existence of neutral RDV is negligible, RDV cation A dominates. At pH = 3.5 the ratio of neutral RDV and RDV cation A is about 2:1. From 1.5 to 3.5, the ratio of neutral RDV increases. As calculated by docking, the RDV cation A binds more tightly to SBE-β-CD than the neutral RDV, resulting in a lower and more favorable binding-free energy. This coincides with the experimental findings. From pH = 1.5 to 3.5, with increasing ratio of neutral RDV, the binding of the macro-state of the RDV to SBE-β-CD decreases, leading to a lower drug loading.

### Docking of RDV to SBE-β-CD elucidates the supersaturation effect

One of the key experimental findings in this study was the supersaturation effect, as indicated in Fig. 2. Due to the pH dependence, drug loading at pH = 1.7 is larger than that at pH = 3.5. However, when we adjusted the pH from 1.7 to 3.5, the remaining drug loading was larger than the direct drug loading at pH 3.5. This indicates that drug supersaturation effect exists.

As discussed in the last section, at pH = 1.7, the RDV cation A dominates, where the positively charged part of RDV is outside the pocket and interacts with the negatively charged sulfobutyl side chain, while at pH = 3.5 the neutral RDV dominates, where RDV was almost fully hidden in the pocket. One of the mechanisms we could think of is that when pH changes from 1.7 to 3.5, RDV’s positively charged tail moves back into the pocket along with the negatively charged sulfobutyl side chain (see the structure of SBE-β-CD in Graphical Abstract), covering the pocket, tending to maintain a bigger drug loading, than in the case pH is set at 3.5, i.e., stabilization of supersaturated solubility, as illustrated in Graphic Abstract.

This hypothesis is waiting to be tested and confirmed and is beyond the scope of this study. The authors are currently investigating this hypothesis by molecular dynamics simulations and will report the results in the subsequent papers.

## Conclusions

In terms of solubilization effect, it was found that phase solubility of RDV increased significantly from intrinsic 1.7 mmol/L at 25 ^o^C to 60 mmol/L at 37 ^o^C in pH = 1.5 solution with increase of SBE-β-CD concentration from 0 to 185 mM. The solubilization could reach equilibrium in as quick as 3 h incubation time. Thermodynamically, the solubilization of RDV in SBE-β-CD is a spontaneous clathration process with negative Gibbs free energy ΔG driving the process toward more stable complexation with increased entropy ΔS. Accurate pKa calculations helped decide the appropriate protonation states at different pH conditions. Specific conformations at corresponding pH conditions were used in the subsequent calculations to correlate with drug loading pH dependence and stabilization of the drug solubility supersaturation.

Altogether, based on accurate QM-based pKa predictions, a novel procedure combining QM, MD, and molecular docking has been explored to account for the experimentally determined macroscopic behavior of remdesivir in SBE-β-CD. It demonstrates the strength of the combination of experimental and mechanism-based computational methods to study complex mechanisms in drug-excipient interactions. Such collaboration could be potentially extended to other dosage forms and has the power of discovering undiscovered lands in the realm of drug formulation and delivery.

## Data Availability

The datasets used and/or analyzed during the current study are available from the corresponding author on reasonable request.
